# Walking the Unseen Path: Black Older Adults, Dementia, and Care in Canada

**DOI:** 10.1002/alz70858_101411

**Published:** 2025-12-24

**Authors:** Ingrid Waldron, Lydia Kapiriri, Ngozi Faith Iroanyah, Maria Belen Miguel, Sadaf Saleem Murad‐Kassam, Lori Letts, Laura Garcia Diaz, Anthea Innis, Pamela Baxter, Letebrhan Ferrow, Oluwagbemiga Oyinlola

**Affiliations:** ^1^ McMaster University, Hamilton, ON, Canada; ^2^ Department of Health, Aging and Society, Faculty of Social Sciences, McMaster University, Hamilton, QC, Canada; ^3^ York University, Toronto, ON, Canada; ^4^ Alzheimer Society of Canada, Mississauga, ON, Canada; ^5^ Alzheimer's Society of Ontario, Toronto, QC, Canada; ^6^ Faculty of Nursing, College of Health Science, University of Alberta, Edmonton, AB, Canada; ^7^ School of Rehabilitation Science, Faculty of Health Sciences, Hamilton, ON, Canada; ^8^ Department of Health, Aging and Society, Faculty of Social Sciences, McMaster University, Hamilton, ON, Canada; ^9^ McGill University, Montreal, QC, Canada

## Abstract

**Background:**

The Alzheimer Society of Canada's report, the *Many Faces of Dementia in Canada* projects a staggering 507% increase in the prevalence of dementia among Black populations in Canada by 2030. However, this alarming statistic underscores a troubling void in empirical research on the lived experiences of Black older adults with dementia within the Canadian context. Existing studies, predominantly from the United States and the United Kingdom, offer limited applicability, as they overlook the distinct multicultural and sociopolitical realities that define Canada. This lacuna in research deprives policymakers and practitioners of the culturally nuanced evidence necessary to design inclusive and effective programs tailored to the needs of Black older adults navigating dementia.

**Method:**

This qualitative study represents the first empirical inquiry into the lived experiences of first‐generation Black older adults with dementia in Canada. Adopting an interpretive narrative methodology, it critically interrogates the interplay of race, culture, and caregiving, with specific focus on mobility. 12 older adults with moderate dementia residing in the Greater Toronto and Hamilton Area (GTHA) were recruited through purposive sampling. Data were gathered via in‐depth interviews and subjected to thematic analysis, thereby elucidating nuanced insights. Knowledge translation measures—including infographics, webinars, and local presentations—have been undertaken to disseminate the findings more widely.

**Result:**

Findings revealed that Black older adults with dementia face substantial challenges navigating both indoor and outdoor spaces, compounded by cultural stigma and societal taboos. Participants struggled with tasks like locating rooms or recalling actions, which fuelled frustration and helplessness. Mobility aids were vital for some, while others retained limited independence indoors. Safety concerns and fear of getting lost often confined individuals to their homes, with outings requiring caregiver supervision. Losing driving privileges further deepened isolation. Cultural stigma framed dementia as a personal or familial failure, discouraging open discussion and delaying support.

**Conclusion:**

The study emphasizes how stigma and fragmented support systems disproportionately impact the autonomy and self‐determination of Black older adults with dementia. Future research should prioritize culturally sensitive care and strengthening of community resources to combat stigma and uphold the dignity of people with dementia in Canada.

